# Shared data science infrastructure for genomics data

**DOI:** 10.1186/s12859-019-2967-2

**Published:** 2019-08-22

**Authors:** Hamid Bagheri, Usha Muppirala, Rick E. Masonbrink, Andrew J. Severin, Hridesh Rajan

**Affiliations:** 1 Department of Computer Science, Iowa State University, 226 Atanasoff Hall, Ames, 50011 USA; 2 Genome Informatics Facility, Iowa State University, 206 Science I, Ames, 50011 USA

**Keywords:** Shared Data Science Infrastructure, Domain-Specific Language, Boa_*g*_, Genome Annotation

## Abstract

**Background:**

Creating a scalable computational infrastructure to analyze the wealth of information contained in data repositories is difficult due to significant barriers in organizing, extracting and analyzing relevant data. Shared data science infrastructures like Boa_*g*_ is needed to efficiently process and parse data contained in large data repositories. The main features of Boa_*g*_ are inspired from existing languages for data intensive computing and can easily integrate data from biological data repositories.

**Results:**

As a proof of concept, Boa for genomics, Boa_*g*_, has been implemented to analyze RefSeq’s 153,848 annotation (GFF) and assembly (FASTA) file metadata. Boa_*g*_ provides a massive improvement from existing solutions like Python and MongoDB, by utilizing a domain-specific language that uses Hadoop infrastructure for a smaller storage footprint that scales well and requires fewer lines of code. We execute scripts through Boa_*g*_ to answer questions about the genomes in RefSeq. We identify the largest and smallest genomes deposited, explore exon frequencies for assemblies after 2016, identify the most commonly used bacterial genome assembly program, and address how animal genome assemblies have improved since 2016. Boa_*g*_ databases provide a significant reduction in required storage of the raw data and a significant speed up in its ability to query large datasets due to automated parallelization and distribution of Hadoop infrastructure during computations.

**Conclusions:**

In order to keep pace with our ability to produce biological data, innovative methods are required. The Shared Data Science Infrastructure, Boa_*g*_, provides researchers a greater access to researchers to efficiently explore data in new ways. We demonstrate the potential of a the domain specific language Boa_*g*_ using the RefSeq database to explore how deposited genome assemblies and annotations are changing over time. This is a small example of how Boa_*g*_ could be used with large biological datasets.

## Background

As sequencing data continues to pile up in the online repositories [1], scientists can increasingly use multi-tiered data to better answer biological questions. A major barrier to these analyses lies with attaining a scalable computational infrastructure that is available to domain experts with minimal programing knowledge. The lengthy time investment required for data wrangling tasks like organization, extraction, and analysis is increasing and is a well-known problem in bioinformatics [2]. As this trend continues, a more robust system for reading, writing and storing files and metadata will be needed.

This can be achieved by borrowing methods and approaches from computer science. Boa_*g*_ is a language and infrastructure that abstracts away details of parallelization and storage management by providing a domain specific language and simple syntax [3]. The main features of Boa_*g*_ are inspired by existing languages for data-intensive computing. These features include robust input/output, querying of data using types/attributes and efficient processing of data using functions and aggregators. Boa_*g*_ can be implemented inside a Docker container or as a Shared Data Science Infrastructure (SDSI). Running on a Hadoop cluster [4], it manages the distributed parallelization and collection of data and analyses. Boa_*g*_ can process and query terabytes of raw data. It also has been

shown to substantially reduce programming efforts, thus lowering the barrier of entry to analyze very large data sets and drastically improve scalability and reproducibility [4]. Raw data files are described to Boa_*g*_ with attribute types so that all the information contained in the raw data file can be parsed and stored in a binary database. Once complete, the reading, writing, storing and querying the data from these files is straightforward and efficient as it creates a dataset that is uniform regardless of the input file standard (GFF, GFF3, etc). The size of the data in binary format is also smaller.

### Domain specific languages and Databases in Bioinformatics

Genomics-specific languages are also common in high-throughput sequencing analysis such as S3QL, which aims to provide biological discovery by harnessing Linked Data [5]. In addition, there are libraries like BioJava [6], Bioperl [7], and Biopython [8] that provide tools to process biological data.

MongoDB is an open source NoSQL database that also supports many features of traditional databases like sorting, grouping, aggregating, indexing, etc. MongoDB has been used to handle large scale semi-structured or NoSQL data. Datasets are stored in a flexible JSON format and therefore can support data schema that evolves over time. MapReduce [9] is a framework that has been used for scalable analysis in scientific data. Hadoop is an open source implementation of MapReduce. In the MapReduce programming model, mappers and reducers are considered as the data processing primitives and and are specified via user-defined functions. A mapper function takes the key-value pairs of input data and provides the key-value pairs as an output or input for the reduce stage, and a reducer function takes these key-values pairs and aggregates data based on the keys and provide the final output. There are organizations that have used the power of MongoDB and Hadoop framework together [10] to address challenges in Big Data. Genomics England [11] runs the 100,000 Genomes Project [12] using MongoDB to harness huge amount of data in bioinformatics. There are also several tools in the field of high-throughput sequencing analysis that use the power of Hadoop and MapReduce programming model. Heavy computation applications like BLAST, GSEA and GRAMMAR have been implemented in Hadoop [13]. SARVAVID [14] has implemented five well-known applications for running on Haddop: BLAST, MUMmer, E-MEM, SPAdes, and SGA. BLAST [15] was also rewritten for Hadoop by Leo *et.al.* [16]. In addition to these programs, there are other efforts based on Hadoop to address RNA-Seq and sequence alignment [17–19].

A significant barrier to utilize the Hadoop framework in bioinformatics is the difficulty of the interface and the amount of expertise that are needed to write a MapReduce programs [20]. The proposed work tries to abstract away details of these complexities and open a door for more bioinformatics application. Most applications could be called from MapReduce rather than reimplementing them. Unfortunately, there currently does not exist a tool that combines the ability to query databases, with the advantage of a domain specific language and the scalability of Hadoop into a Shared Data Science Infrastructure for large biology datasets. Boa_*g*_, on the other hand is such a tool but is currently only implemented for mining very large software repositories like GitHub and Sourceforge. It recently has been applied to address potentials and challenges of Big Data in transportation [21].

### Potential for data parallelization framework in biology

There are several very large data repositories in biology that could take advantage of a biology specific implementation of Boa_*g*_*:* The National Center for Biotechnology Information (NCBI), The Cancer Genome Atlas (TCGA), and the Encyclopedia of DNA Elements (ENCODE). NCBI hosts 45 literature/molecular biology databases and is the most popular resource for obtaining raw data for analysis. NCBI and other web resources like Ensembl are data warehouses for storing and querying raw data, sequences, and genes. TCGA contains data that characterizes changes in 33 types of cancer. This repository contains 2.5 petabytes of data and metadata with matched tumor and normal tissues from more than 11,000 patients. The repository is comprised of eight different data types: Whole exome sequence, mRNA sequence, microRNA sequence, DNA copy number profile, DNA methylation profile, whole genome sequencing and reverse-phase protein array expression profile data. ENCODE is a repository with a goal to identify all the functional elements contained in human, mouse, fly and worm. This repository contains more than 600 terabytes (personal communication with @EncodeDCC and @mike_schatz) of data with more than 40 different data types with the most abundant data types being ChIP-Seq, DNase-Seq and RNA-Seq. These databases represent only the tip of the iceberg of potential large data repositories that could benefit from the Boa_*g*_ framework. While it is common to download and analyze small subsets of data (tens of Terabytes for example) from these repositories, analyses on the larger subsets or the entire repository is currently computationally and logistically prohibitive for all but the most well-funded and staffed research groups. While BioMart [22], Galaxy, and other web-based infrastructures provide an easy to use tool for users without any knowledge in programming to download subsets of the data, the needs of the advanced users using the entire database aren’t met as evidenced by a plethora of bash scripts, R scripts and Python scripts that are widely utilized and reinvented by bioinformaticians. Retrieving the genomics data and performing data-intensive computation can be challenging using existing APIs. Biomartr [23] is an R package to retrieve raw genomics data that tries to minimize some of this complexity.

Here we discuss an initial implementation of Boa for genomics on a small test dataset, NCBI Refseq, a database containing data and metadata for 153,848 genome annotation files (GFF). We show the potential of Boa_*g*_ in a comparative context with python and MongoDB by assessing various statistics of the Refseq database and answer the following four questions.

- What is the smallest and largest genome in RefSeq? How has the average number of exons per gene in genomes of a clade changed for genomes deposited before and after 2016?
- How has the popularity of the top five assembly programs in bacteria changed over time?
- How has assembly quality changed for genomes deposited before and after 2016?

## Results

### Summary statistics of RefSeq

While it is straightforward to use the RefSeq website (https://www.ncbi.nlm.nih.gov/refseq/) to look up this information for your favorite species, it is cumbersome to look up this information for tens to hundreds species. Similarly, while each of these genomes have an annotation file, querying and summarizing information contained in this annotation file from several related genomes such as average number of genes, average number of exons per gene and average gene size requires downloading and organizing the annotation files of interest prior to calculating the statistics.

Data from the RefSeq database was downloaded, a schema was designed and a Hadoop sequence file generated for use with Boa_*g*_, a domain specific language and shared data infrastructure. The RefSeq data used in this first implementation of Boa_*g*_ contains GFF files and metadata from bacterial (143,907), archaea (814), animal (480), fungal (284) and plant (110) genomes. Each genome has metadata related to the quality of its assembly (Genome size, scaffold count, scaffold N50, contig count, contig N50), the assembler software, and the genic data contained within the GFF annotation file.

Our goal is to implement Boa_*g*_ on a biological dataset to demonstrate a means to explore large datasets. In the following subsections, we will answer the four questions posed in the introduction and explore Boa_*g*_ efficiency in storage, speed, and coding complexity.

### What is the largest and smallest genome in RefSeq?

As of February 16th, 2019, the largest genome in the RefSeq database was *Orycteropus afer afer* (aardvark, GCF_000298275.1) at a length of 4,444,080,527 bp. The smallest genome is RYMV, a small circular viroid-like RNA hammerhead ribozymein sequenced from Rice and annotated as a Rice yellow mottle virus satellite (viruses). Its complete genome has a length of 220 bases and has a RefSeq id GCF_000839085.1.

**Fig. 1:**
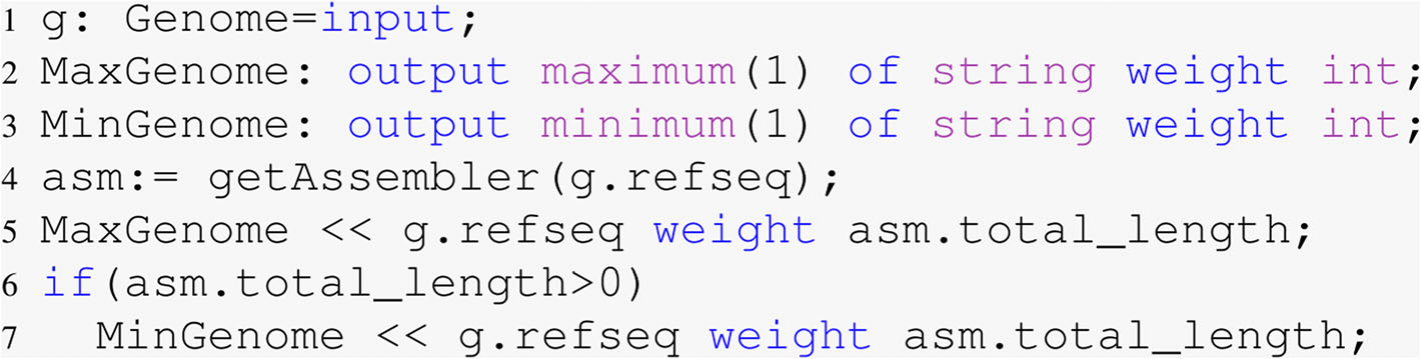
Code to find the smallest and largest genomes in RefSeq

With the full RefSeq dataset in a Hadoop sequence file, this statistic only required seven lines of Boa_*g*_ code (Fig. 1). In line one, variable g is defined as a Genome which is a top-level type in our language. MaxGenome and MinGenome are output aggregators that produce the maximum and minimum genome length respectively. Lines five and seven in the code emit the assembly total length to the reducer for all the genomes in the dataset, then the reducer will identify the largest and smallest genomes. It took Boa_*g*_ approximately 30 seconds to finish this query when using a single node without Hadoop. It took the equivalent query using python approximately one hour using a single core.

#### How has the average number of exons per gene in a species clade changed for genomes deposited before and after 2016?

Due to the rapid advancement of sequencing technologies and genome assembly/annotation programs, any meaningful biological changes in gene and exon frequency will be confounded with these advancements. We explored seven clades: five kingdoms and two phyla to explore how exon number, gene number, gene length and exons per gene have changed before and after 2016. These branches of the tree of life included Bacteria, Archaea, Fungi, Ascomycota (a fungal phylum), Viriplantae (plants), Eudicotyledons (a clade in flowering plants) and Metazoans (a clade of animals). In the last two years, the number of sequenced bacterial genomes has nearly quadrupled, while all other clades have seen at least a 50% increase in RefSeq database (Tables 1 and 2). The number of genes, number of exons and exons per gene have increased for all clades database (Tables 1 and 2). Since prokaryotes do not have exons, Bacteria and Archaea were excluded from this query for exon number and exon per gene (NA). A higher number of exons per gene for the Eukaryotes suggests that gene models are improving and becoming less fragmented. This improvement could be due to improvements in gene annotation software or assembly contiguity.

**Table 1:** Exon Statistics for years > = 2016

Name	Total species	Exon number	Gene number	Gene Length	Exon per Gene
Bacteria	92,287	N/A	4*.*3 *k ±* 1*.*5 *k*	890 *±* 64	N/A
Fungi	90	32*.*3 *k ±* 1*.*8 *k*	10 *k ±* 3*.*5 *k*	1*.*6 *k ±* 171	2*.*9 *±* 1*.*3
Archaea	338	N/A	2*.*9 *k ±* 0*.*9 *k*	851 *±* 31	N/A
Viridiplantae	46	385 *k ±* 155 *k*	43 *k ±* 21 *k*	4*.*1 *k ±* 1*.*3 *k*	9*.*2 *±* 1*.*9
Metazoas	185	462 *k ±* 280 *k*	24*.*9 *k ±* 10*.*3 *k*	23 *k ±* 11*.*8 *k*	17*.*7 *±* 6*.*4
Ascomycota	70	28*.*4 *k ±* 13*.*7 *k*	10*.*4 *k ±* 3*.*1 *k*	1*.*6 *k ±* 142	2*.*5 *±* 0*.*8
eudicotyledons (dicots)	37	397 *k ±* 167 *k*	45 *k ±* 22 *k*	3*.*8 *k ±* 688	9 *±* 1*.*3

**Table 2:** Exon Statistics for years < 2016

Name	Total species	Exon number	Gene number	Gene Length	Exon per Gene
Bacteria	51,537	N/A	3*.*8 *k ±* 1*.*5 *k*	885 *±* 65	N/A
Fungi	194	29 *k ±* 20 *k*	9*.*2 *k ±* 3*.*5 *k*	1*.*6 *k ±* 254	2*.*8 *±* 1*.*5
Archaea	474	*N/A*	2*.*9 *k ±* 0*.*8 *k*	855 *±* 40	N/A
Viridiplantae	61	273 *k ±* 153 *k*	32 *k ±* 17 *k*	4*.*1 *k ±* 2*.*3 *k*	8 *±* 2*.*5
Metazoas	262	314 *k ±* 211 *k*	22*.*3 *k ±* 9*.*6 *k*	22 *k ±* 12 *k*	13*.*4 *±* 5*.*4
Ascomycota	143	25*.*2 *k ±* 14*.*3 *k*	9*.*5 *k ±* 3*.*1 *k*	1*.*6 *k ±* 205	2*.*4 *±* 1
eudicotyledons (dicots)	41	328 *k ±* 133 *k*	38 *k ±* 16 *k*	4 *k ±* 1*.*4 *k*	8*.*6 *±* 1*.*3

We find fewer genes in archaea than in bacteria, at 2.9k and 4.3k genes respectively. The highest gene numbers in eukaryotes are plants (43k), with animals and fungi being having fewer genes at 24.9k and 10k, respectively [24]. However, the mean gene length for these clades has not changed between timepoints, indicating that the increased exon content per gene is likely due to an improvement in annotation software.

**Fig. 2:**
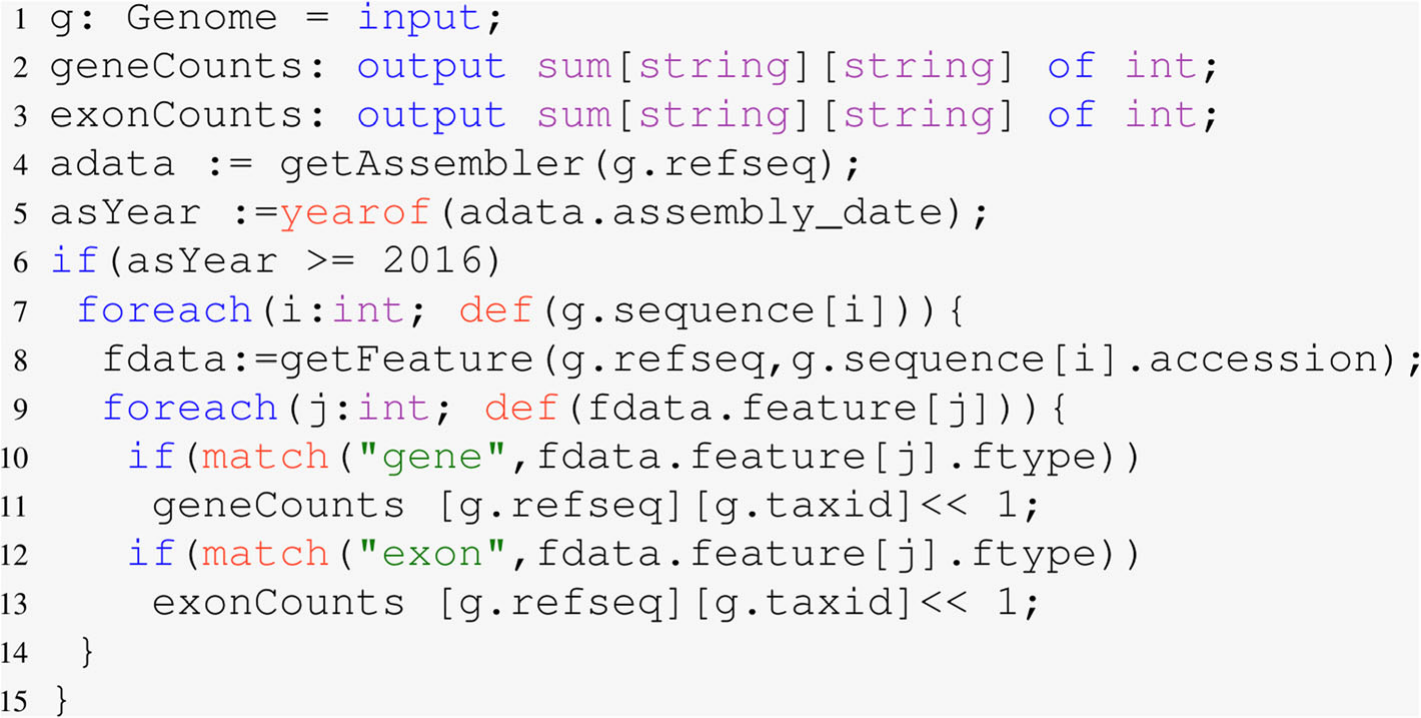
Number of exons, genes, and exons per gene after 2016. The output is shown in Table 1

This query required 15 lines of Boa_*g*_ code (Fig. 2) using a five node shared Hadoop cluster on Bridges with 64 mappers approximately 42 minutes to answer this question. It took the equivalent query using 45 lines of python code approximately 20 hours using a single core.

### How has the popularity of bacterial genome assembly programs changed?

**Fig. 3:**
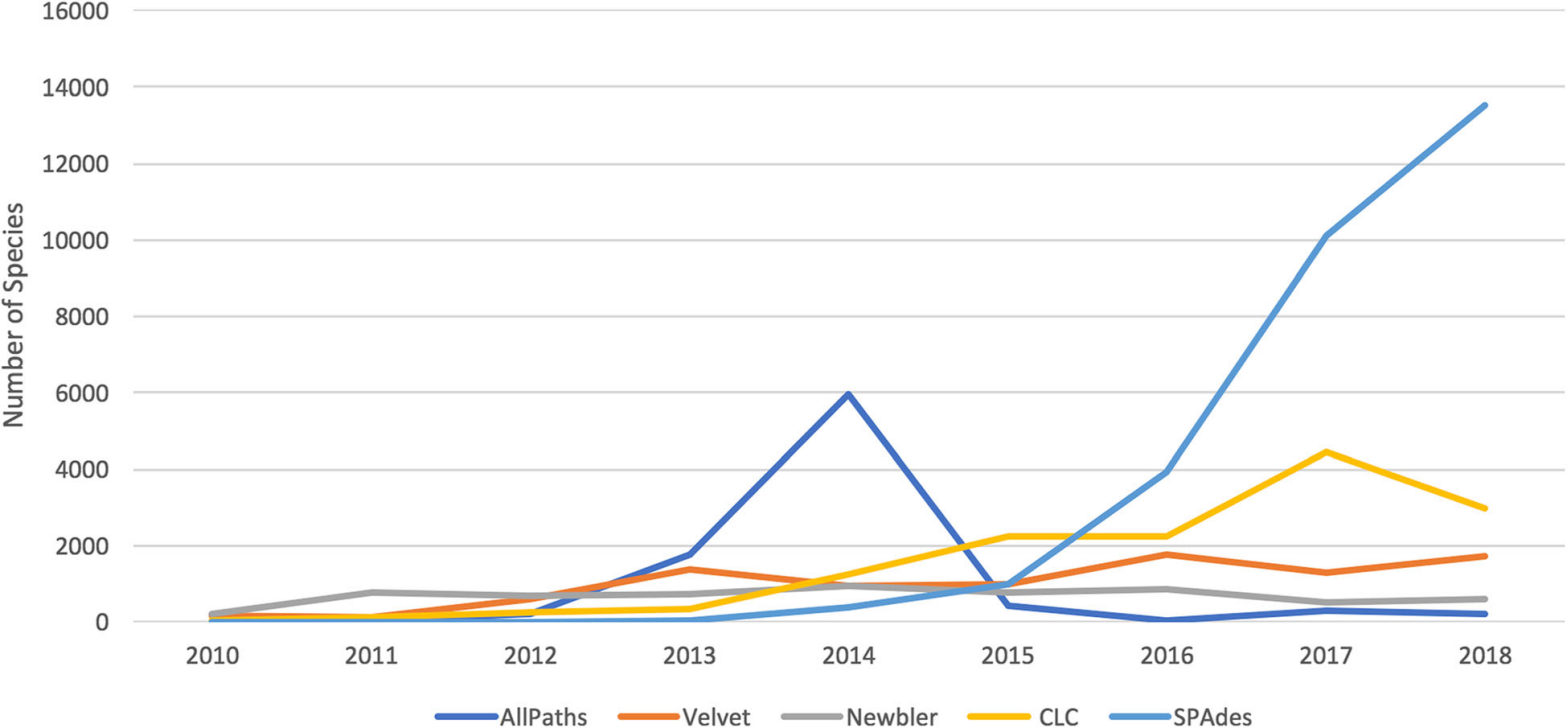
Bacterial assembly programs popularity over time. The output of this script is shown in Fig. 4

The choice of genome assembly program to assemble a genome depends on many factors including but not limited to user familiarity of the program in the domain, ease of use, assembly quality, turnaround time. Looking at the number of genomes assembled by the top five most popular assemblers in bacteria indicate that more genomes are being assembled over time, that there was a brief period of popularity with AllPaths in 2014, and a rapid rise in popularity of the SPAdes assembler in the last couple of years. CLC workbench offers a GUI interface to users without programming experience, and has consistently maintained a slice of the user market (Fig. 3).

**Fig. 4:**
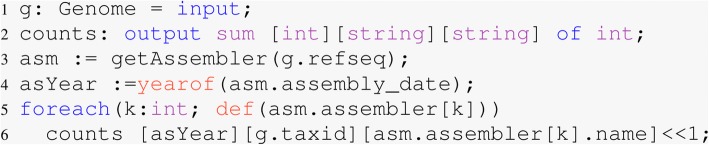
Assembler programs for Bacteria over the years

This query required six lines of Boa_*g*_ code Fig. 4 using a five node Hadoop cluster with 32 mappers approximately 30 seconds to answer this question. The equivalent single-cored python query took approximately one hour with 35 lines of code.

### How has metazoan assembly quality changed for genomes deposited before and after 2016?

To minimize bias in organismal variation and assembly software, we have limited our comparison to metazoans and the top three assembly programs. The popular assembly programs for metazoans has been AllPaths after 2016 while SOAPdenovo was the most popular one before 2016. A high-quality assembly is characterized by a low scaffold count and high N50, stats that dramatically improved at the 2016 transition. As it can be seen in Tables 3 and 4, the scaffold count has decreased for all three assemblers after 2016 while the contig N50 metric has increased. This is not a surprise, as assembly algorithms are expected to improve over time. Newbler had a dramatic decrease in scaffold count after 2016. The highest average N50 among metazoans belongs to AllPaths.

**Table 3:** List of top three most used assembly programs for Metazoa (Year > =2016)

Kingdom	Program Name	species	Total length	Scaffold-count	ScaffoldN50	ContigCount	ContigN50
Metazoa	SOAPdenovo	21	1B ± 0.8B	38 *k ±* 49 *k*	7.8 M ± 11 M	86 k ± 66 k	98 k ± 208 k
	AllPaths	48	0.9B ± 0.7B	7.1 k ± 7 k	4.3 M ± 1.4 M	33 k ± 38 k	188 k ± 335 k
	Newbler	7	0.8B ± 0.9B	3.3 k ± 2.2 k	877 k ± 910 k	56 k ± 80 k	75 k ± 60 k

**Table 4:** List of top three most used assembly programs for Metazoa (Year < 2016)

Kingdom	Program Name	species	Total length	Scaffold-count	ScaffoldN50	ContigCount	ContigN50
Metazoa	SOAPdenovo	98	1*.*2*B ±* 0*.*7*B*	40 *k ±* 38 *k*	4*.*5 *M ±* 13 *M*	116 *k ±* 79 *k*	42 *k ±* 48 *k*
	AllPaths	54	1*.*5*B ±* 1*.*1*B*	11 *k ±* 13 *k*	7*.*4 *M ±* 9*.*7 *M*	119 *k ±* 97 *k*	38 *k ±* 32 *k*
	Newbler	18	0*.*9*B ±* 0*.*9*B*	87 *k ±* 117 *k*	2*.*1 *M ±* 2*.*3 *M*	133 *k ±* 157 *k*	34 *k ±* 27 *k*

**Fig. 5:**
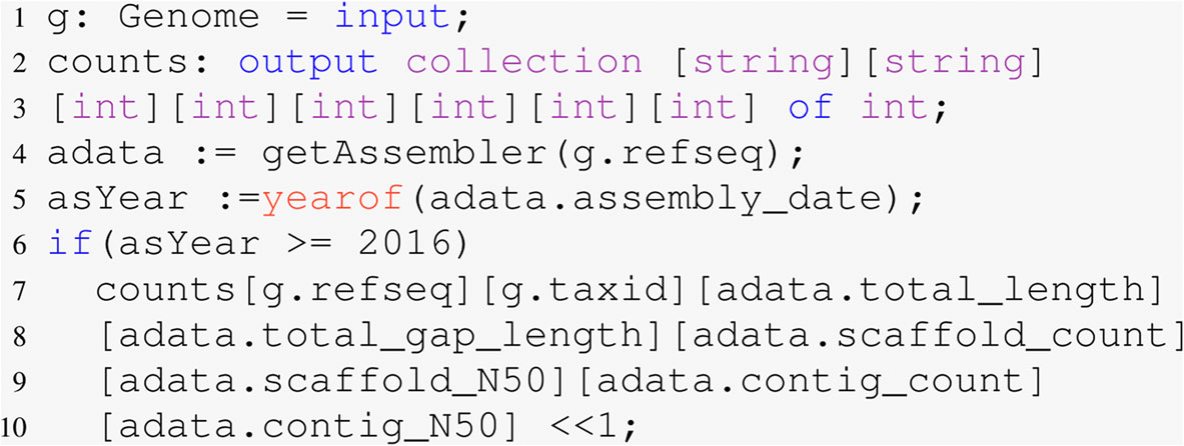
Assembly statistics for genomes for years after 2016. The output is shown in Table 5

This query required 10 lines of Boa_*g*_ code using five nodes Hadoop cluster with 32 mappers approximately 30 seconds. An equivalent single-cored Python query took approximately one hour and 32 lines of code (Fig. 5).

## Discussions

### Database storage efficiency and computational efficiency with Hadoop

**Fig. 6:**
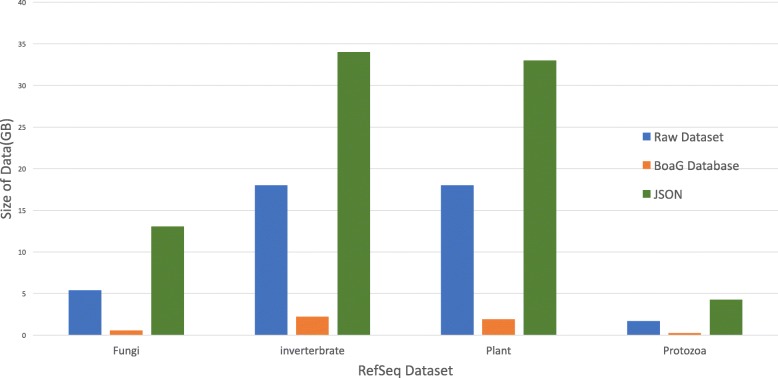
The Boa_*g*_ database size comparison with the raw data in the RefSeq as well as the JSON version of the dataset

One benefit of the Boa_*g*_ database is the significant reduction in required storage of the raw data. The downloaded NCBI RefSeq data was 379GB, but reduced to 64GB (6.2 fold reduction) in the Boa_*g*_ database. This data size reduction is due to the binary format of Hadoop Sequence file which makes disk writing faster than a text file (Fig. 6). A fungi-only subset of the RefSeq data was dramatically reduced from 5.4GB to 0.5 GB (10 fold reduction). This variability in size reduction is presumably due to variability in the number and size of files among phyla.

**Fig. 7:**
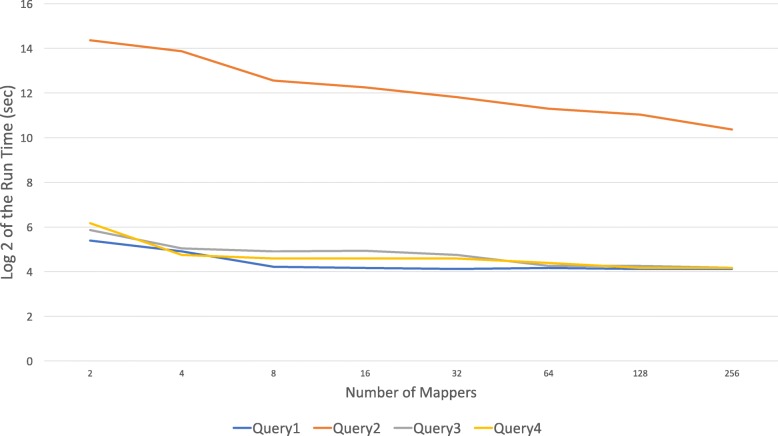
Scalability of Boa_*g*_ programs (time is in Log base 2 (sec)). Queries 1,2,3 and 4 are the four questions investigated here

**Fig. 8:**
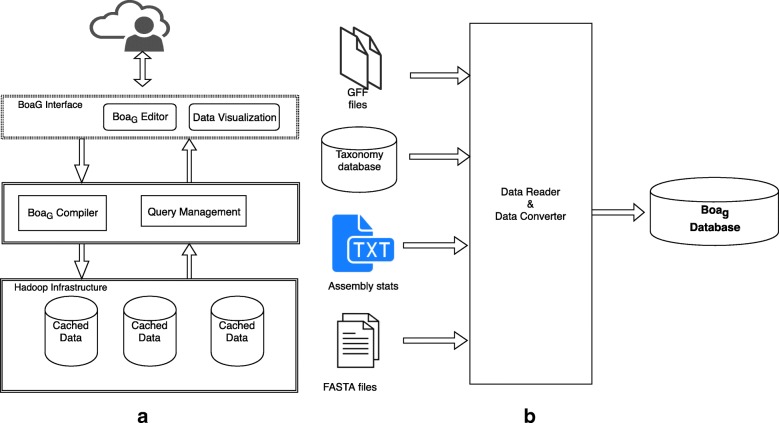
Boa_*g*_ Architecture and Data Generation

A second benefit of Boa_*g*_ is its ability to take advantage of parallelization and distribution during computation. Increasing the number of Hadoop mappers for a Boa_*g*_ job decreases the query turnaround time. Taking the four queries we posed in the introduction, we varied the level of Hadoop mappers to show the speedup that results by adding additional Hadoop mappers to an analysis. Figure 7, demonstrates the exponential decrease in required computation time with a corresponding increase in the number of Hadoop mappers. As you can see, if the number of mappers are not optimized for the amount of computational infrastructure than the second query takes approximately 350 minutes on 2 mappers to complete. However, as more mappers are added, the time required levels out to less than one minutes on assembly related queries. This lower bound of this relationship is presumably due to the overhead of splitting and gathering of data across the mappers. As we add more mappers the running time decreases for example with 256 mappers runtime is 22 minutes on the entire RefSeq. It is not difficult to see the benefit of using a domain specific language like Boa_*g*_ and Hadoop infrastructure to query much larger biological datasets than RefSeq (Fig. 8).

Taking advantages of Hadoop based infrastructure, all the queries in the Tables 5 and 6 that describe the genome assembly statistics before and after 2016 transition required less than a minute.

**Table 5:** Kingdoms and average summary statistics for their genome assemblies (Years > =2016)

Tax ID	Name	Species	Total length	Scaffold-count	ScaffoldN50	ContigCount	ContigN50
2	Bacteria	92,290	4*.*3 *M ±* 1*.*6 *M*	66 *±* 78	0*.*9 *M ±* 1*.*4 *M*	132 *±* 176	0*.*39 *M ±* 0*.*86 *M*
4751	Fungi	90	29 *M ±* 15 *M*	139 *±* 159	1*.*3 *M ±* 0*.*9 *M*	360 *±* 688	0*.*78 *M ±* 1 *M*
2157	Archaea	338	2*.*9 *M ±* 0*.*98 *M*	52 *±* 40	0*.*38 *M ±* 0*.*43 *M*	74 *±* 121	0*.*53 *M ±* 71 *M*
33,090	Viridiplantae	46	0*.*97*B ±* 0*.*88*B*	9*.*1 *k ±* 18*.*3 *k*	31 *M ±* 49 *M*	38 *k ±* 43 *k*	1*.*8 *M ±* 4*.*9 *M*
33,208	Metazoas	185	1*.*2*B ±* 0*.*95*B*	20*.*6 *k ±* 43*.*7 *k*	22 *M ±* 36 *M*	53 *k ±* 77 *k*	2*.*5 *M ±* 7*.*9 *M*
71,240	eudicotyledons (dicots)	37	0*.*91*B ±* 0*.*76*B*	6*.*4 *k ±* 10*.*6 *k*	26 *M ±* 50 *M*	40 *k ±* 44 *k*	1*.*6 *M ±* 4*.*3 *M*

**Table 6:** Kingdoms and average summary statistics for their genome assemblies (Years <= 2015)

Tax ID	Name	Species	Total length	Scaffold Count	ScaffoldN50	ContigCount	ContigN50
2	Bacteria	51,962	3*.*8 *M ±* 1*.*6 *M*	45 *±* 82	1*.*3 *M ±* 1*.*5 *M*	126 *±* 177	0*.*27 *M ±* 0*.*55 *M*
4751	Fungi	202	2*.*9 *M ±* 17 *M*	341 *±* 699	2 *M ±* 1*.*7 *M*	858 *±* 1433	0*.*55 *M ±* 0*.*75 *M*
2157	Archaea	470	29 *M ±* 1 *M*	17 *±* 16	1*.*35 *M ±* 1*.*17 *M*	110 *±* 126	0*.*38 *M ±* 0*.*7 *M*
33,090	Viridiplantae	67	0*.*62*B ±* 0*.*68*B*	22*.*9 *k ±* 46*.*6 *k*	14*.*7 *M ±* 24*.*9 *M*	52*.*5 *k ±* 71*.*6 *k*	0*.*47 *M ±* 1*.*8 *M*
33,208	Metazoas	295	1*.*3*B ±* 1*B*	37*.*4 *k ±* 64*.*2 *k*	7*.*2 *M ±* 14 *M*	118*.*6 *k ±* 119 *k*	0*.*13 *M ±* 1*.*2 *M*
71,240	eudicotyledons (dicots)	46	0*.*754*B ±* 0*.*750*B*	26*.*3 *k ±* 53*.*5 *k*	17 *M ±* 27 *M*	58*.*8 *k ±* 74 *k*	0*.*3 *M ±* 1*.*6 *M*

### Comparison between MongoDB and Boa_*g*_

**Fig. 9:**
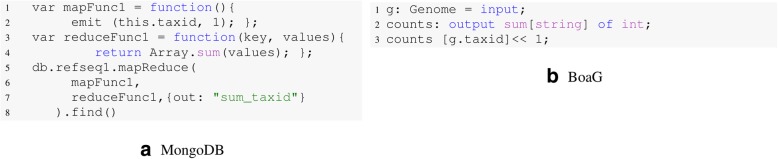
Comparison of the code needed to query the number of assembler programs per taxon id run on Refseq Data. On the left side, the MongoDB code needs eight lines of code in Python whereas the BoaG script needs only three lines of code. **a**. MongoDB query to calculate number of assembler programs per taxon id. **b**. Equivalent Boag query needs fewer lines of code

An analysis in Boa_*g*_ requires fewer lines of codes than other languages available like MongoDB and Python (Fig. 9). The file size in the Boa_*g*_ database is much smaller than the JSON file used in MongoDB, as Boa_*g*_ utilizes a binary format. Since the data schema in MongoDB also needs to be saved along with the data, the output files are larger and take longer to write (Fig. 6). The JSON file size is larger and on average it is more than double size of the RefSeq raw data. While experts in MongoDB may write this query more efficiently, the Boa_*g*_ language requires fewer lines of code (Fig. 9), thereby providing an easier interface for bioinformaticians to explore big data.

The performance of MongoDB and Hadoop has been previously compared [25], showing that the read-write overhead of Hadoop has a lower read-write overhead (Table 7).

**Table 7:** Comparison between MongoDB and BoaG

Feature	MongoDB	BoaG
Lines of Code	larger	smaller because it abstracts details of data analysis
Data generation time	longer due to the larger file	faster because of Binary file
Data file	JSON is 2.7 times larger than raw data	Hadoop Sequence file 5 times smaller than raw data
Schema Flexibility	Yes. Supports semi-structured data	Yes. Schema and compiler can be modified
MapReduce	Yes	Yes

### Comparison between Python and Boa_*g*_

**Fig. 10:**
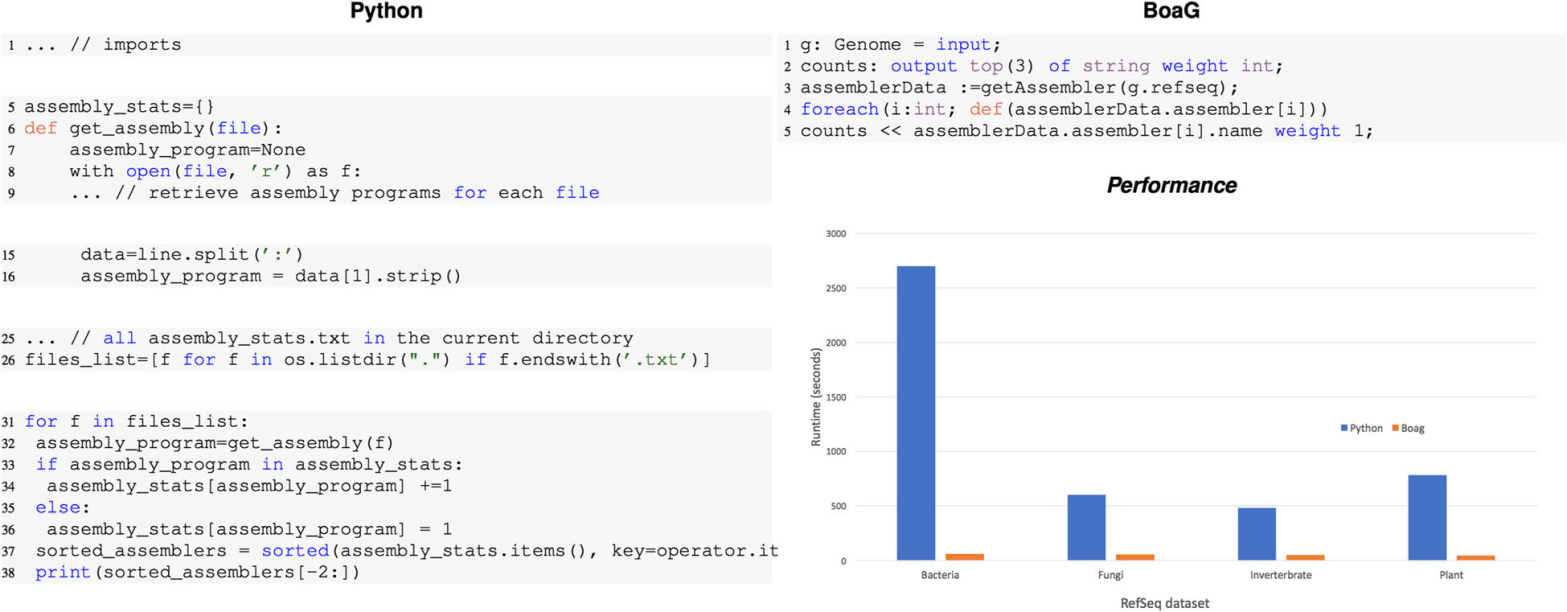
Comparison of Line of Code (LOC) and performance to answer query “ What are the top three most used assembly programs?” run on Refseq Data. On the left side, the equivalent Boa_g_ code needs 38 lines of code in Python whereas the Boa_g_ script needs only five

A general-purpose language like Python could also be utilized to execute the same queries investigated here. However, the Python code would be larger and require learning how to use Python libraries. To illustrate, we wrote an example program in Python to calculate the top three most used assembly programs required only five lines of code in Boa_*g*_ language. In Python, a similar analysis required 38 lines of code (Fig. 10). Because Python needs to aggregate the output data, it needs more lines of code and a longer runtime. This advantage inherent to domain-specific languages will speed up a researcher’s ability to query large datasets.

**Fig. 11:**
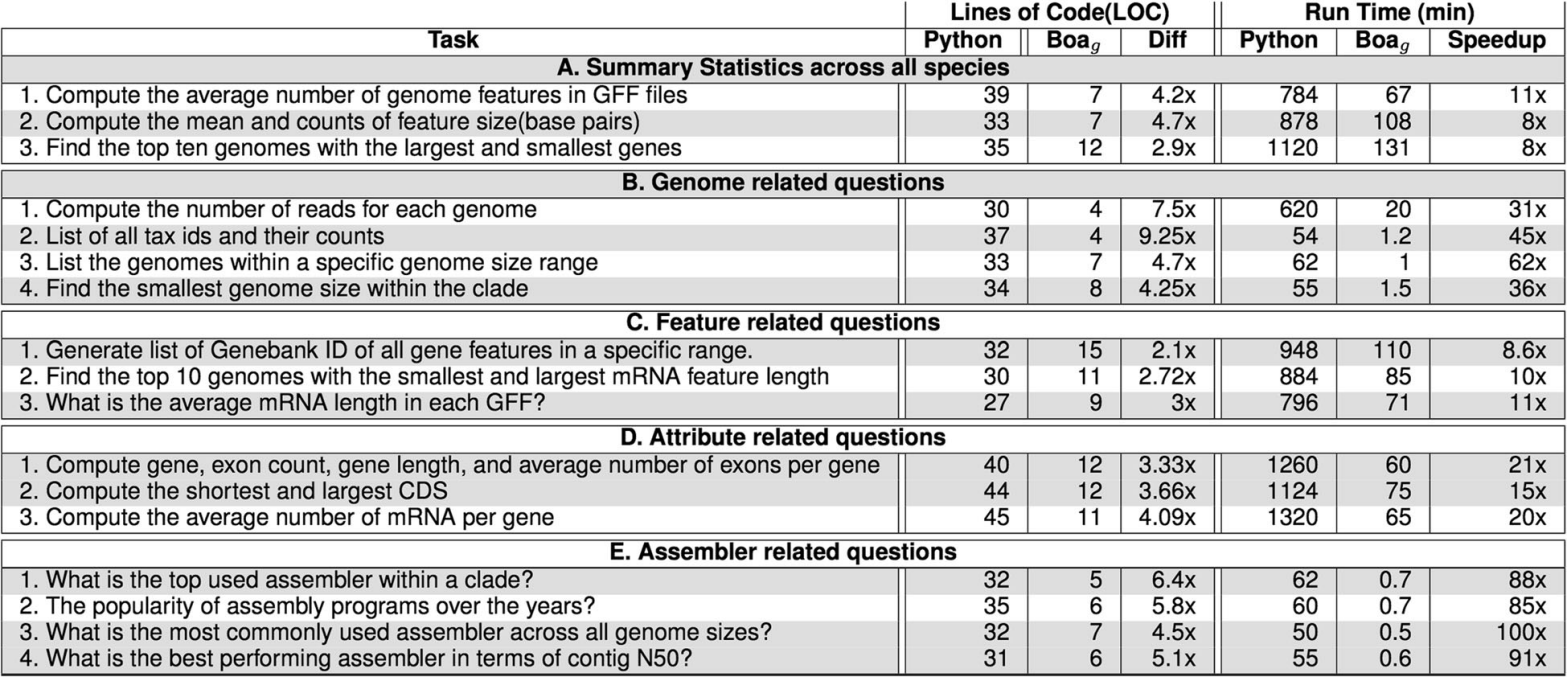
Example of Boa_g_ programs to compute different tasks on the full RefSeq dataset. The python programs were running on the single core. The Hadoop infrastructure on Bridges has 5 shared nodes with 32 mappers. While these queries can be written in parallel in python, this needs more lines of code and more programming skills to write a parallel code

More comparisons in terms of runtime and lines of codes are given in Fig. 11. These tests were performed on an iMac system with processor 4 GHz Intel Core i7 and 32 GB 1867 MHz DDR3 of memory.

Boa_*g*_ also provides an external implementation that allows users to bring their own implementation from Python, Perl, Bash, etc. Not all users of the infrastructure can run any arbitrary scripts on the infrastructure. Scripts need to be converted to a DSL function so that they will not cause security issues for the infrastructure.

## Conclusion

In this work, we presented Boa_*g*_ which is a domain-specific language and shared data science infrastructure that takes advantage of Hadoop distribution for large-scale computations. Boa_*g*_ ‘s infrastructure opens the exploration of large datasets in ways that were previously not possible without deep expertise in data acquisition, data storage, data retrieval, data mining, and parallelization. The RefSeq database was used as an example dataset from Biology to show how to implement the domain-specific language Boa_*g*_ for biological discovery. Boa_*g*_ is able to query the RefSeq dataset in under 2 minutes for most queries, offering a substantial time savings from other methods. Many examples, tutorials, and a Docker container are available a GitHub repository. This paper provides a proof of concept behind the Boa_*g*_ infrastructure and its ability to scale to much larger datasets. This is the first step towards providing a shared data science infrastructure to explore large biological datasets.

In future, we will integrate new data types including the Non-Redundant protein database, biological ontologies, SRA, etc. We will also update the Boa_*g*_ database and provide a publicly available web-interface for researchers to run query on our infrastructure.

## Methods

### Choice of Biological repository for prototype implementation

RefSeq is a relatively small dataset containing information on well-annotated sequences spanning the tree of life: plants, animals, fungi, archaea and bacteria. The smaller database size permits rapid iterations of Boa_*g*_ applied to biology, and illustrates the benefits of a genomics specific language. RefSeq also has a decent amount of metadata about genome assemblies and their annotations for which as far as we know has not been explored as a whole. Unfortunately, due to the rapid advancement of sequencing technologies and genome assembly/annotation programs, deriving biologically meaningful information from comparisons of assembly stats across the entire dataset is not possible. However, as a demonstration of the usefulness of a Boa_*g*_ infrastructure, we show how straightforward it is to ask questions about how the database and the metadata has changed over time which gives insight into how improvements in sequencing technology and assembly/annotation programs have affected the data contained in this repository. These types of information would be challenging to procure directly from the online repository.

### Design and implementation considerations

As a domain specific language careful consideration must be taken in its design for Hadoop based infrastructure implementation for RefSeq data. The overall workflow for Boa_*g*_ requires a program written in Boa_*g*_ that is submitted to the Boa_*g*_ infrastructure (Fig. 8 (a)). The infrastructure takes the submitted program and compiles with the Boa_*g*_ compiler and executes the program on a distributed Hadoop cluster using a Boa_*g*_ formatted database of the raw data. Boa_*g*_ has aggregators, which are functions that run on the entire or a large subset of the database to take advantage of the Boa_*g*_’s database, which is designed to distribute both data and compute across a Hadoop cluster.

### A Boa_*g*_ infrastructure provides the following benefits for exploring large datasets

- A computational framework on top of Hadoop that can query large dataset in minutes.
- An efficient data schema that provides storage efficiency and parallelization.
- An expandable database integration.
- A domain-specific language that can be incorporated in a container, Galaxy framework or along with any language like R or Python in a Juypter notebook.

#### Genomics-specific Language and data schema

To create the domain-specific language for biology in Boa_*g*_, we created domain types, attributes and functions for the RefSeq dataset that includes the following raw file types: FASTA, GFF and associated metadata, as shown in Table 8, Genome, Sequence, Feature, and Assembler are types in Boa_*g*_ language and taxid, refseq, etc are attributes of the genome type. We created the data schema based on the Google protocol buffer, which is an efficient data representation of genomic data that provides both storage efficiency and efficient computation for Boa_*g*_.

**Table 8:** Domain types for Genomics data in BoaG

Type	Attributes	Details
Genome	taxid	Taxonomy ID of each species
	refseq	Refseq ID of the GFF file
	Sequence	List of sequence reads in each GFF file [26].
	AssemblerRoot	List of assembly programs associated with this genome
	accession	Accession number
Sequence	header	Header of Sequence
	FeatureRoot	List of features including exon,gene,mRNA, and CDS associated with this sequence
	seq	Actual DNA sequences from FASTA files
FeatureRoot	refseq	This field shows the key ID
	feature	This field is the list of features associated with this ID
Feature	accession	Accession code of the Sequence
	seqid	Sequence ID
	source	A text qualifier that describes the algorithm or procedure that generated this feature.
	ftype	Type of the feature
	start	starting point of the feature
	end	End point of the feature
	score	Score of the feature. This is a floating point number.
	strand	+ and - for positive and negative strand respectively
	phase	Phase of the feature. The phase is one of the integers 0, 1, or 2
	Attribute	List of attributes for each feature
	parent	Shows the parent of the attribute
Attribute	id	Attribute ID
	tag	Attribute tag including gbkey etc.
	value	Value of the tag
AssemblerRoot	Assembler	List of assembly programs
	total-length	Total length or genome size (base pair)
	total-gap-length	Total gap length after genome assembly
	scaffold-N50	Scaffold N50 metric
	scaffold-count	Scaffold count metric
	contig-N50	Contig N50 metric
	contig-count	Contig count metric
Assembler	name	Assembly program used to assemble the genome
	desc	Program attributes: program name, program version, etc.

#### Output Aggregators in Boa_g_

Table 9 shows the predefined aggregators in the Boa_*g*_ language for example top, mean, maximum, minimum, etc. These aggregators are also available in traditional RDBS and MongoDB [27], however Boa_*g*_ is flexible enough to define new aggregators. Boa_*g*_ provides a specific type called output types that collect and aggregate data and provide a single result. When a Boa_*g*_ script is running in parallel, it emits values to the output aggregator that collects all data and provides the final output. Aggregators also can contain indices that would be a grouping operation similar to traditional query languages.

**Table 9:** The BoaG aggregators list

Aggregator	Description
MeanAggreagtor	Calculates the average
MaxAggreagtor	Finds the maximum value
SumAggregator	Calculates the sum of the emitted values to the reducer
MinAggregator	Finds the minimum value
TopAggregator	Takes an integer argument and returns the top elements for the given argument
StDevAggregator	Calculates the standard deviation

### Boa_*g*_ database and new data type integration

The Boa_*g*_ infrastructure is designed to fully utilize data parallelization facilities in Hadoop infrastructure. The raw data for file types and metadata was parsed into a Boa_*g*_ database on top of a Hadoop sequence file (Fig. 8 (b)). A compiler, file reader, and converter were written in Java to generate this database and are provided in the GitHub repository (https://github.com/boalang/bio/tree/master/compiler). In order to integrate new dataset the data schema in protocol buffer format needs to be modified and a data reader in Java that reads the raw data, for example GFF, TXT, Fastq, etc, is needed that can convert it to a binary format of Boa_*g*_ database. An additional example is provided in the GitHub repository.

Boa_*g*_ efficiency was tested on a shared Hadoop cluster on Bridges with 5 nodes and up to 256 map tasks.

### Data availability

All scripts, step by step process of scientific discovery, and additional examples of Boa queries used in this paper can be found in our repository. The raw data files, Boa_*g*_ database and JSON MongoDB files can be obtained from an online repository (https://boalang.github.io/bio/). A Docker container with Boa_*g*_ scripts, a Boa_*g*_ sequence file of a subset of the raw files and instructions on how to use Boa_*g*_ can also be downloaded from this location. We have generated a subset of GFF files and assembly statistics files for all fungi data contained in RefSeq. This data subset is 5.4 GB and can be used to test Boa_*g*_ queries for reproducible results.

### Run Boa_*g*_ on Docker container and Jupyter

For the fungal data subset, users can run a containerized version of a 3 node Hadoop cluster for Boa_*g*_ as well as Jupyter versions on a single machine. These integrations with current technologies can help users test and run queries and reproduce our results. Instructions on how to run a Docker version and a Jupyter version of Boa_*g*_ are available on this website: https://boalang.github.io/bio/.

### Application of Boa_*g*_ to the RefSeq database

A total of 153,848 annotations (GFF), assembly (FASTA) files, and metadata were downloaded from NCBI RefSeq [28] and written to a Boa_*g*_ database. Metadata included genome assembly statistics (Genome size, scaffold count, scaffold N50, contig count, contig N50) and assembler software used to generate the assembly from which the genome annotation file was created.

## Data Availability

Boa_g_ compiler’s source code, documentation, Docker container, etc are provided on the GitHub repository (https://github.com/boalang/bio) The Boa_g_ website is located here (https://boalang.github.io/bio/).

## Abbreviations

Boa_g_: Boa for Genomics
DSL: Domain-Specific Language
RefSeq: Reference sequence database
SDSI: Shared Data Science Infrastructure
